# Gender mainstreaming in sweetpotato breeding in Uganda: a case study

**DOI:** 10.3389/fsoc.2023.1233102

**Published:** 2023-12-15

**Authors:** Reuben T. Ssali, Sarah Mayanja, Mariam Nakitto, Janet Mwende, Samuel Edgar Tinyiro, Irene Bayiyana, Julius Okello, Lora Forsythe, Damalie Magala, Benard Yada, Robert O. M. Mwanga, Vivian Polar

**Affiliations:** ^1^International Potato Center (CIP-SSA), Kampala, Uganda; ^2^School of International Development, University of East Anglia, Norwich, United Kingdom; ^3^National Agricultural Research Laboratories (NARL), National Agricultural Research Organization (NARO), Kampala, Uganda; ^4^National Crops Resources Research Institute (NaCRRI), National Agricultural Research Organization (NARO), Kampala, Uganda; ^5^Natural Resources Institute (NRI), University of Greenwich, Chatham Maritime, United Kingdom; ^6^Mukono Zonal Agricultural Research and Development Institute (MUZARDI), National Agricultural Research Organization (NARO), Kampala, Uganda; ^7^International Potato Center (CIP), Lima, Peru

**Keywords:** sweetpotato, Uganda, gender mainstreaming, plant breeding, value chain actors

## Abstract

**Purpose:**

In Uganda, sweetpotato [*Ipomoea batatas* (L.) Lam] is typically a “woman’s crop,” grown, processed, stored and also mainly consumed by smallholder farmers for food and income. Farmers value sweetpotato for its early maturity, resilience to stresses, and minimal input requirements. However, productivity remains low despite the effort of breeding programs to introduce new varieties. Low uptake of new varieties is partly attributed to previous focus by breeders on agronomic traits and much less on quality traits and the diverse preferences of men and women in sweetpotato value chains.

**Method:**

To address this gap, breeders, food scientists, and social scientists (including gender specialists) systematically mainstreamed gender into the breeding program. This multidisciplinary approach, grounded in examining gender roles and their relationship with varietal and trait preferences, integrated important traits into product profiles.

**Results:**

Building on earlier efforts of participatory plant breeding and participatory varietal selection, new interventions showed subtle but important gender differences in preferences. For instance, in a study for the RTBFoods project, women prioritized mealiness, sweetness, firmness and non-fibrous boiled roots. These were further subjected to a rigorous gender analysis using the G+ product profile query tool. The breeding pipelines then incorporated these gender-responsive priority quality traits, prompting the development of standard operating procedures to phenotype these traits.

**Conclusion:**

Following an all-inclusive approach coupled with training of multidisciplinary teams involving food scientists, breeders, biochemists, gender specialists and social scientists, integration into participatory variety selection in Uganda enabled accentuation of women and men’s trait preferences, contributing to clearer breeding targets. The research has positioned sweetpotato breeding to better respond to the varying needs and preferences of the users.

## 1 Introduction

### 1.1 Why sweetpotato breeders in Uganda paid attention to gender

In Uganda, sweetpotato [*Ipomoea batatas*, (L) Lam] is typically considered a “woman’s crop” grown by smallholder farmers, about 60% of whom are women (Zawedde et al., 2014; Echodu et al., 2019). Farmers value sweetpotato for its early maturity, resilience to biotic and abiotic stresses, and minimal input requirements. Sweetpotato thrives in dry areas with poor soils, so the crop appeals to women who are often allocated marginal lands of the household’s farmland. Aside from subsistence production, women also process sweetpotato into secondary products like chips, flour and other value-added products. Women also generate and control income from sale of sweetpotato vines and surplus roots. Women are responsible for household food preparation. As such, they value sweetpotato for bridging the hunger gap since it matures much earlier in the season than the other crops and for its high energy density. Additionally, sweetpotato, especially orange-fleshed type, has a soft texture and sweet taste which appeals to children and is thus useful as a weaning food (Hagenimana et al., 2001). Sweetpotato leaves are increasingly eaten as a vegetable or a relish, contributing micronutrients (Mudege et al., 2017) and diversifying the diets. The crop residues are fed to small animals and ruminants; further endearing sweetpotato to women given their responsibility in animal feeding (Dione et al., 2015).

Breeding efforts have focused on producing new varieties that responded to farmers’ needs. By 2016, the Ugandan breeding program had released 22 sweetpotato varieties – both local and improved (Mwanga et al., 2001, 2003, 2007, 2009, 2011, 2016; Grüneberg et al., 2015). The released varieties were selected mainly for yield, weevil resistance, dry matter content, virus resistance and nutrition, especially for vitamin A, with less attention to the gender-differentiated needs of farmers and other value chain actors. Generally, breeders selected for agronomic traits instead of the culinary and processing traits preferred by men and women. For example, the size and shape of sweetpotato roots which largely determine the effort women exert in food preparation were hardly considered. Consequently, released varieties have not been widely adopted, with only 6.9% of the harvest area under improved varieties (Thiele et al., 2021) by women and men partly because their needs were not considered in plant breeding. As a result, farmers, especially women, still rely on landraces whose seed degenerates more quickly compared to improved seed due to pest and diseases infestation (Zawedde et al., 2014; Ogero et al., 2023). Also, Uganda has more than 900 local landraces, which are adapted to the very diverse local production conditions, making it difficult for the improved varieties to replace them (Yada et al., 2010; Labarta et al., 2012). Reliance on landraces is, in part, due to women’s limited access to new knowledge, skills and immobility (Puskur et al., 2021). This is compounded by limited access to input and output markets as Katungi et al. (2018) report for similar crops. Women are also expected to commence with cultivating the family land, and only till their own land later in the season. They thus experience time poverty, a challenge that is exacerbated by their triple roles (production, reproduction, caregiving) and contributing to their limited access to improved varieties.

This case study documents the evolution in including gender perspectives in the sweetpotato breeding program in Uganda. It highlights the initial gender gaps, the response to (mostly donor-driven) demands to consider gender in breeding through participatory varietal selection (PVS) and the strong incorporation of gender into the breeding programs through targeted project interventions coupled with gender training. This evolution resulted in greater gender consciousness among breeders, leading to a more gender-responsive breeding program in Uganda.

Elsewhere, breeding programs have endeavored to integrate a gender lens in their plans. In South Africa, the maize breeding programs were ready to incorporate gender responsive traits but lacked guidance on what these specific traits could be because on-farm trials were unable to predict gendered differences in trait preferences. The suggested solution was to review agronomic practices used by female plot managers at advanced stages of the breeding pipeline (Cairns et al., 2022). For beans in Kenya, a combination of gender-disaggregated participatory varietal selection (PVS) and choice experiments identified short cooking time as a “must have” gender-neutral trait (Katungi et al., 2018). So, breeding programs invested in routine evaluation of bean cooking time, using a Mattson cooker. In Tanzania, a gender yield gap was identified where women registered much lower yields than men. This was attributed to their triple roles; exacerbated by limited access to education; technology, training, and minimal land rights (Nchanji et al., 2020). Resultantly, bean breeders were guided to incorporate training on good agricultural practices when introducing new varieties to control selection bias. The Consortium of International Agricultural Research Center (CGIAR) scientists note that promoting socially equitable varieties like nutritionally improved varieties risks creating a yield penalty compared to the best agronomic varieties which could reduce their adoption (Kholová et al., 2021). Therefore, breeders need to work with policy makers to ensure that the yield gap does not affect adoption of such socially advantageous varieties.

This case study is organized as follows. In section 1.2, we delve into the study context and highlight organizations and actors involved, the geographical scope of the breeding program, the size and composition of the breeding team as well as the chief characteristics of target beneficiaries. Section 2 describes the case study methodology while section 3 analyses the evidence of gender integration in sweetpotato breeding in Uganda. Section 4 assesses the approaches, and outcomes of gender integrations in sweetpotato breeding. Section 5, wraps up our discussion of the good practices, lessons learned and recommendations.

### 1.2 Context

**Figure 1:**
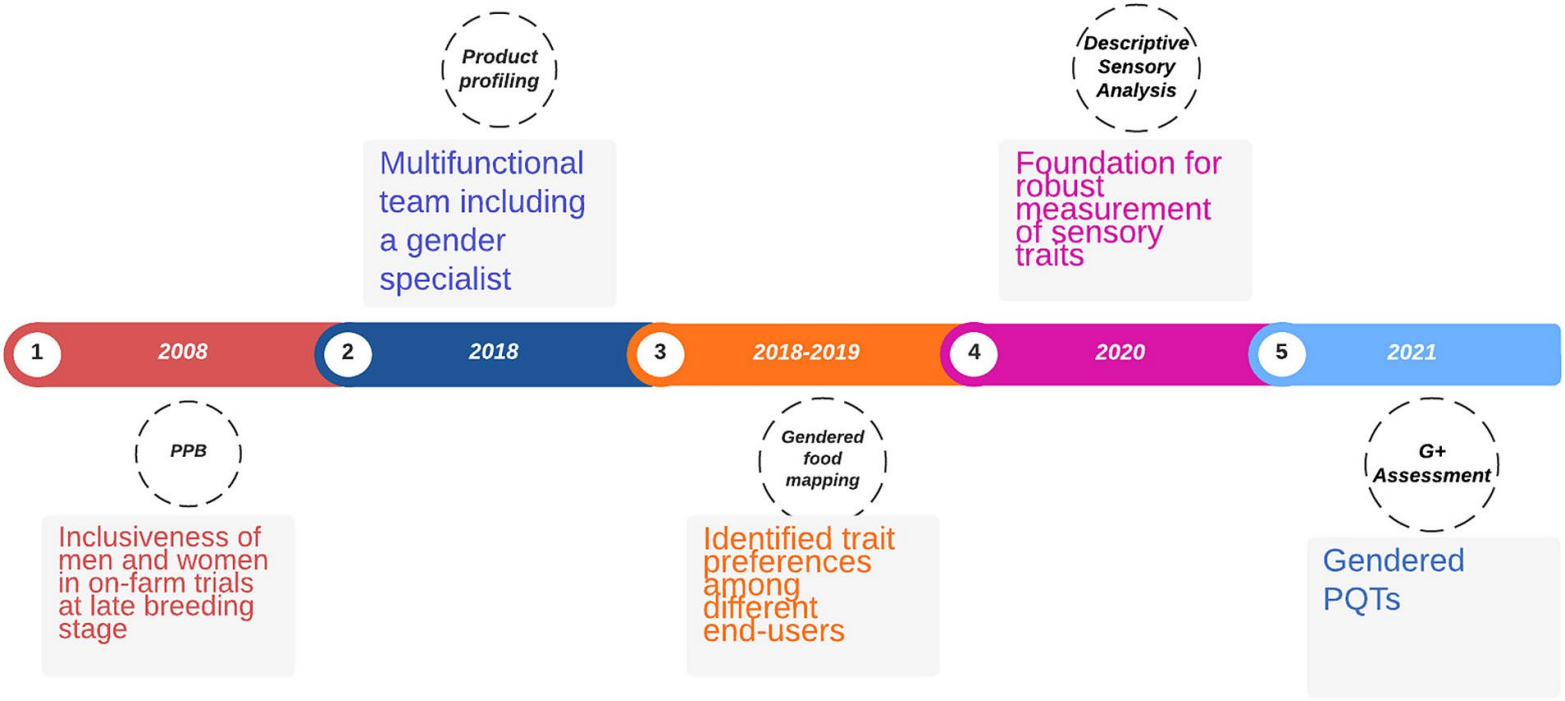
Key events and decisions incorporating gender into sweetpotato breeding in Uganda.

The sweetpotato breeding program in Uganda has evolved over the years (Figure 1). It was established to generate an expanded range of sweetpotato cultivars resistant to the high sweetpotato virus disease (SPVD) and sweetpotato weevil pressures in East and Central Africa, combining nutrition, cooking quality and high yield. More recently, breeding for end-user preference with a gender perspective has come into focus with the increased appreciation of the salient gender needs, roles and responsibilities in sweetpotato cropping systems. End-users in this perspective include seed multipliers, producers, traders, processors, consumers as well as value chain support services.

**Figure 2:**
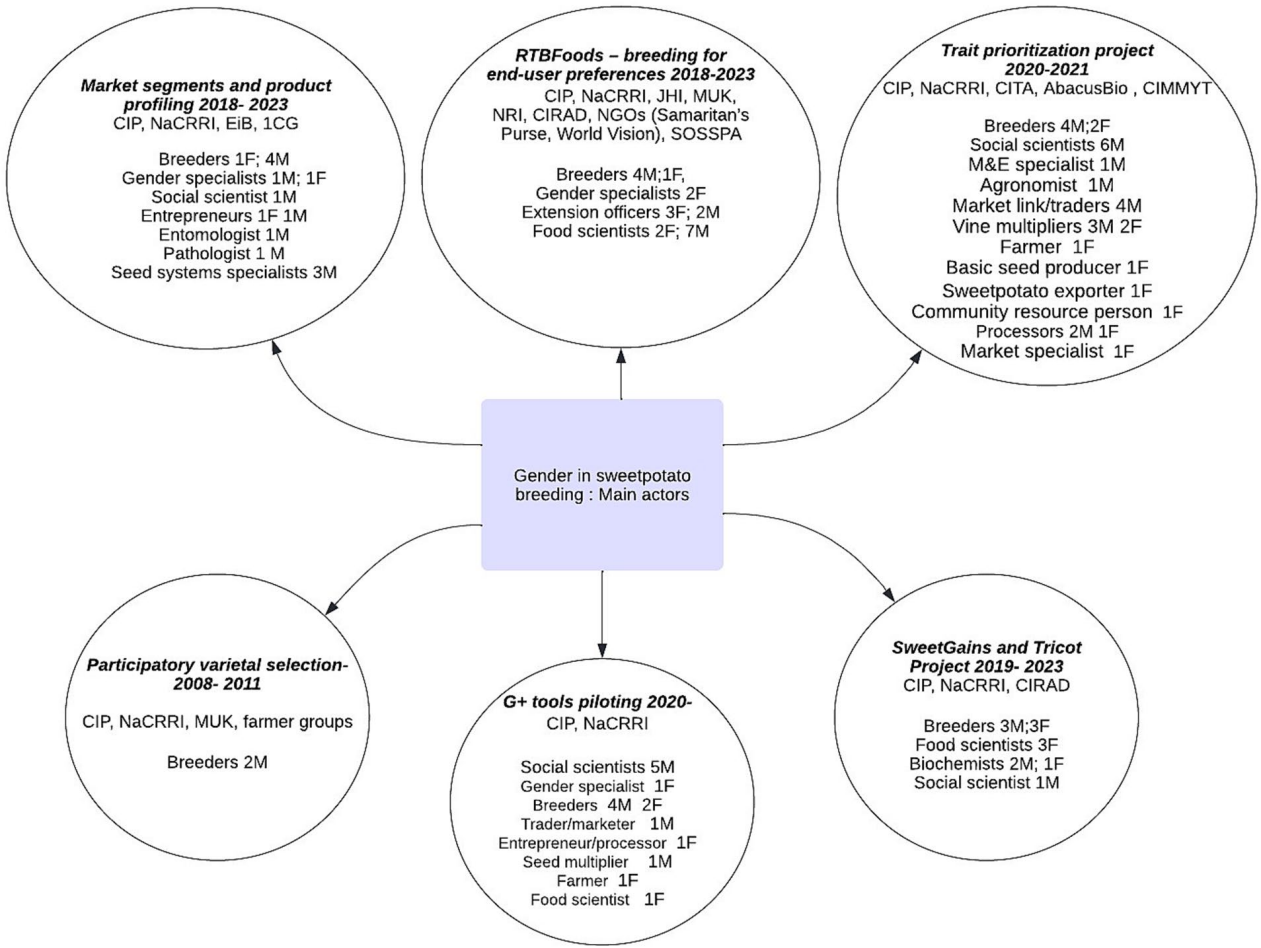
Project collaborators and research team composition (F-female and M-male) engaged over time to incorporate gender in sweetpotato breeding in Uganda.

The International Potato Center (CIP) and the National Crops Resources Research Institute (NaCRRI), an institute of Uganda’s National Agriculture Research Organization (NARO), have collaborated with local and international partners in implementing the various research projects (Figure 2). Local partners included Makerere University, international NGOs (World Vision and Samaritan’s Purse), and farmer organizations such as the Soroti Sweetpotato Producers and Processors Association (SOSPPA). Partnerships were formed with international organizations: UK’s James Hutton Institute (JHI), Natural Resources Institute (NRI) and AbacusBio, France’s Centre de Coopération Internationale en Recherche Agronomique pour le Développement (CIRAD), Spain’s Centro de Investigación y Tecnología Agroalimentaria de Aragón (CITA) as well as other CGIAR centers such as the International Maize and Wheat Improvement Center (CIMMYT). The activities were supported by funding from CGIAR platforms, like the Excellence in Breeding (EiB) which produced the G+ toolkit (Ashby and Polar, 2021; CGIAR, 2021). The CGIAR Research Program on Roots, Tubers and Bananas (RTB) facilitated work on the triadic comparison of technologies (tricot). Other important funding was from The Bill and Melinda Gates Foundation for the RTBfoods and Sweetpotato Genetic Advances and Innovative Seed Systems (SweetGAINS) projects. The United States Agency for International Development (USAID) and United Kingdom’s Foreign, Commonwealth and Development Office also supported projects like Development and Delivery of Biofortified Crops at Scale (DDBIO). Over time the sweetpotato program engaged breeders, food scientists, biochemists, gender specialists, social scientists, and data scientists to systematically and holistically understand how to mainstream gender into breeding. These collaborations strengthened the technical and infrastructural capacity, making it possible to address gendered preferences of sweetpotato characteristics. The RTBFoods project, for example, developed methods for measuring sensory traits (e.g., taste, mealiness, firmness) preferences of different consumer segments, thus facilitating gender integration in trait selection (Figure 2).

The geographical coverage of the program stretched across all of Uganda’s nine agro-ecological zones with activities in Kabale, Mpigi, Kabarole, Kamuli, Iganga, Busia Mbale, Kumi, Moroto, Lira, Kitgum, Adjumani, Arua, Kamwenge, Luwero and Hoima districts. The breeding program targeted actors across the value chain including sweetpotato farmers (mainly smallholders with <1 acre plot size), small-medium scale processors (mostly women and the youth), traders (mostly female retailers and male wholesalers) and consumers (rural, peri-urban, and urban) (Mayanja et al., 2019).

## 2 Case study methodology

Step 1. At conception we pooled information on all breeding research projects that tackled inclusiveness of other stakeholders in the breeding process. Each of these projects were reviewed considering: (1) Why the research was conducted, (2) Who comprised the research team, (3) when and how the research was conducted, (4) what the findings of the study were and how they influenced subsequent projects and sweetpotato breeding specifically.

Step 2. A workshop of a transdisciplinary team comprising of three social scientists, two food scientists, two gender specialists and two breeders, was conducted to discuss and document the approaches and lessons learnt during the implementation of the various projects. Insights from this workshop were used to write this case study using the guide developed under the project titled ‘Elaboration of case study integrating gender into breeding objectives and decision’ (Supplementary material 1).

## 3 Evidence of gender integration in sweetpotato breeding

### 3.1 Resources and other sources of information on gender generated

**Figure 3:**
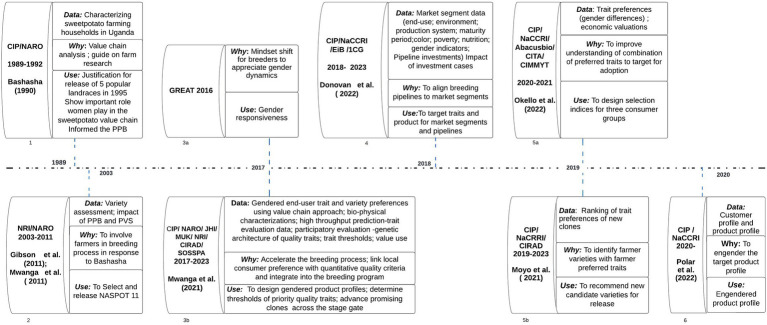
Timeline of gendered research activities in sweetpotato breeding.

A timeline of gender research in sweetpotato breeding shows that before 1989 breeding was largely biological with little focus on gender (Figure 3). A diagnostics study between 1989 and 1992 by the sweetpotato research team identified the important roles women play across the value chain (Bashaasha et al., 1995; Figure 3, 1). The study highlighted the need to involve end-users in breeding, motivating breeders to introduce participatory plant breeding (PPB) and PVS with groups of women and men farmers in Central Uganda. However, in PPB and PVS (from 2003 to 2011) though the breeders worked closely with farmers in the selection process; little attention was given to urban consumers (Figure 3, 2). Also, some specific reasons behind preferences for the variety were unclear, especially because some traits only appealed to certain actors. For example, across the value chain, only farmers participating in the trial would appreciate a variety’s resistance to SPVD (Okello et al., 2022). In 2009, a gender scientist was recruited in the Sweetpotato Action for Security and Wealth in Africa (SASHA) project to support gender mainstreaming in breeding as well as other project components. This contributed to the adoption of a Ugandan bred OFSP variety ‘*Kabode*’ across the borders due to its good eating qualities. Although preferences of men and women processors, producers and consumers were recognized by both CIP and NARO, appropriate tools to allow for refinement and targeting of key traits, market segments, climate uncertainty, changing markets and urbanizing populations and incorporating all the traits in a cultivar remained a challenge. Having identified the important roles of women in the value chain, and recruitment of a gender scientist integration was expected to increase drastically, but this did not happen immediately (Mwanga et al., 2021). This could be attributed to the increased focus on breeding for vitamin A-rich, orange-fleshed sweetpotato (OFSP) in the early 2000’s. Consumer education for women and children on the nutritional benefits of OFSP overshadowed considerations of gender relations affecting adoption of new varieties, despite evidence that women played a leading role in decisions to adopt OFSP (Gilligan et al., 2014).

The five- year RTBfoods project (Figure 3, 3b), which started in 2017, introduced a five-step method to develop food product profiles: a detailed description of a food product, including traits for its ingredients, processing suitability and sensory characteristics such as appearance, taste, texture, and aroma. This is only a subset of the target product profile used by breeding programs to describe the mix of basic traits essential to the success of the market variety and value-added traits being targeted in a new product (variety). The objective of developing a food product profile was to identify and rank the most important characteristics by various food chain actors for consideration as breeding targets. Food product profiles identified end-user preferences with a gender perspective (Forsythe et al., 2021). The five steps were: (1) audit of the state of knowledge, (2) gendered food product mapping, (3) participatory processing diagnosis (PPD), (4) consumer testing of a selected set of four sweetpotato varieties in rural and urban segments, and (5) triangulation of data with alternative methods for G+ food product profile consolidation. This 5-step method gave a prominent leadership role to the gender researcher and the food scientist. This change was intended to regulate the usually predominant role of the breeder in product design.

Under the same project, the state of knowledge (SoK) output (2018) was led by a gender specialist in collaboration with a food scientist who compiled the desirable characteristics of boiled sweetpotato among men and women end-users, based on a literature review and key informant interviews. These characteristics were probed further in a subsequent study using individual interviews (IIs) and focus group discussions (FGDs) of the gendered food mapping. This was also led by the gender specialist with a team comprising food scientists, agronomists and social scientists. The gendered food mapping identified (1) the most and least preferred sweetpotato varieties, (2) end-user preferences at various product stages: raw, during processing and boiled (Mwanga et al., 2021), and (3) champion processors. The study highlighted socio-cultural aspects of livelihoods, land use and ownership, decision making, control of income in the household and labor, among others.

The PPD was conducted in 2019 and was led by a food scientist working with a gender specialist and a social scientist. A selection of four sweetpotato varieties (including most and least preferred varieties) were used for the PPD. Preferences of women processors were determined for raw roots, at each processing step and the final boiled sweetpotato.

Consumer studies to test boiled sweetpotato varieties in rural and urban areas (2019) were led by a food scientist, working with the gender specialist and social scientist. Respondents were monadically presented with cooked samples of local and improved samples of sweetpotato to evaluate in a randomized order. The study sought to (1) understand preferences of consumers; disaggregated by sex, age, income, education level; and (2) identify eating quality attributes penalized by consumers. This marked the end of the RTBFoods Project gender responsive studies.

In 2017, two more initiatives were introduced. First the Gender Responsive Researchers equipped for Agricultural Transformation (GREAT) program was launched in Uganda. Second, the Excellence in Breeding (EiB) Platform introduced the concept of breeding product profile development (Tawanda Reginold Mashonganyika, 2018). Both these initiatives required convening a multifunctional design team including a gender research specialist. GREAT involved capacity building and hands on research which identified gender differentiated trait preferences in production and marketing (Figure 3, 3a).

The EiB approach identified the market leading varieties (for potential replacement) and proposed ‘must-have’ and ‘value-added’ traits, Figure 3, 4. CIP and NARO developed the first sweetpotato product profile with guidance from two gender specialists. Some of the traits put forward included vigorous vine establishment (because women struggled to get planting material) and aroma (most of the new varieties were lacking in characteristic sweetpotato aroma). Nonetheless, these traits were not prioritized in the initial product profile proposed in 2018, as their potential to contribute to the likelihood of replacing the market leading variety was not clearly understood and therefore not appreciated.

The AbacusBio trait prioritization project (Figure 3, 5a), in 2020 and 2021, also contributed to gender responsive sweetpotato breeding. The project was led by a social scientist and involved breeders, a gender scientist, root producers, transporters, retailers, processors, and consumers. It identified three groups of sweetpotato end-users based on categories of trait patterns and direction of preference ranks within each group, i.e., productive output, plant robustness and root quality. There were more women in the root quality group, which gave high preference ranking to dry matter, flesh color, sweetness, skin smoothness and root size. This could be because women are more involved in buying and preparing their household’s food, so they pay attention to root quality. A selection index was developed for each group, and this will facilitate breeding for women-preferred traits.

The triangulation and consolidation of the food product profile in 2021 was led by a food scientist with a team that included a gender specialist, social scientists, breeders, traders and processors. The analysis revealed that (1) a large, hard, sweet root with a smooth skin was considered a good raw sweetpotato, which should be easy to peel and non-fibrous and (2) the boiled or steamed sweetpotato should be sweet, firm, mealy, with a characteristic sweetpotato aroma and non-fibrous.

As part of this process gender and livelihoods assessments were conducted for each of the quality traits in the food product profile, using an adapted version of the G+ product profile query tool (Ashby and Polar, 2019, 2021; CGIAR, 2021) (Figure 3, 6). Some traits like ‘smooth skin’ ‘root size’, ‘sweetness’, ‘firmness’, ‘mealiness’ and the ‘sweetpotato smell’, had the potential to increase the commercial value of the sweetpotato crop. This would attract men to dominate the production and trading of the crop, which could cause gender inequalities, which should be addressed during the release and promotion of these varieties. We thus recommended ‘amend’ or ‘proceed with caution’ for these traits, and suggested strategies that should accompany release of the varieties to maintain women’s active role in sweetpotato markets. As a starting point for deploying the gender-responsive food product profile, five priority quality traits (PQTs) (mealiness, sweetness, firmness, characteristic sweetpotato smell and non-fibrousness) were selected for trait dissection and development of phenotyping protocols (Dufour et al., 2023). A sensory panel was set up and trained to develop a lexicon for descriptive sensory analysis (DSA) as described by Nakitto et al. (2022). Despite being a low-throughput method, DSA enabled the screening of clones for women-preferred quality traits.

Additionally, several proof-of-concept studies identified potential biochemical and biophysical techniques for measuring the gender-responsive PQTs. Spectra and image analysis were explored as high-throughput predictors of the PQTs. The breeding program combined DSA with consumer testing to determine the desirable threshold of these crucial gender-responsive traits. The plot size for the early breeding stages was subsequently increased from 1 m^2^ to 10 m^2^ to provide enough roots for cooking quality analysis. The trait dictionary was also updated to encompass quality traits.

The Triadic comparisons of technologies (TRICOT) is a crowdsourced citizen science methodology. It involves the distribution of a pool of agricultural technologies in different combinations of three to individual farmers. The farmers use and observe these technologies under farm conditions and rank their performance. TRICOT was piloted to assess consumer perceptions of sweetpotato varieties. The study was conducted by a team led by food scientists and included breeders, biochemists, gender specialists and social scientists in 2020 (Moyo et al., 2021; Figure 3, 5b). Its integration into participatory variety selection enabled accentuation of women and men’s trait preferences, contributing to clearer breeding targets. This holistic approach coupled with capacity development gained from the GREAT project and strong institutional support has positioned sweetpotato breeding to better respond to the varying needs and preferences of the users.

### 3.2 How attention to gender has influenced sweetpotato breeding in Uganda

Several aspects of the breeding program have changed with the shift toward gender-responsiveness. The changes include:

- Definition of markets or end users to be targeted currently includes trait preferences for various end-users across the value chain are now considered
- The breeding objectives have been expanded to include gender responsive PQTs
- Breeding methods have been extended to include tools for medium to high throughput phenotyping of gender responsive PQTs like instrumental texture analysis, artificial intelligence, spectral analysis
- Desirable thresholds (targets) for PQTs were established, e.g., using the established instrumental texture analysis SOP, optimal firmness is indicated by test clones that require a minimum of 3700G force to compress
- Gender-responsive selection index has been developed considering weights for PQTs
- Tricot approach (citizen science) ranking preferences for advanced clones by many consumers/producers has been adopted
- Multi-functional teams have been established for joint decision-making during product advancement, not just the breeder, using a production calendar to ensure data to guide the advancement of clones across the breeding pipeline.

During the research, some opportunities arose that made it feasible to integrate gender trait preferences into breeding. For instance, CIP and NARO implemented projects like RTBfoods and SweetGAINS that aimed at modernizing core breeding operations. These contributed to identifying trait preferences by men and women, supporting the development of phenotyping protocols for priority quality traits and acquiring laboratory equipment to enable high throughput phenotyping PQTs. Sweetpotato research scientists benefited from gender-responsive training by both GREAT and RTBFoods projects. These trainings increased the appreciation for gender research and enabled the team to design more inclusive breeding projects. Also, most of the desirable traits included in the gender-intentional product profile to guide the breeding were available within the germplasm. Cross-institutional partnerships involving CIP, NaCRRI, NaRL, FANEL, JHI, Makerere University and CIRAD expanded access to technical expertise, protocols, and laboratory equipment. The product profile design and the product advancement process benefited from creating multi-functional teams between CIP and NARO. In 2021 the sweetpotato breeding programs at CIP and NARO were assessed by the breeding program assessment tool (BPAT) assessors. The assessment revealed several areas for improvement to drive higher rates of genetic gain and adoption in sweetpotato breeding. This provided justification for institutional support to address changes required for gender-responsive research.

The CIP sweetpotato breeder and gender specialist endeavored to bring different stakeholders on board to implement activities as a team. CIP encouraged breeders to lead the process of integrating gender and incorporating the gender results. This process enabled the breeders to appreciate the need to integrate gender over time. Sweetpotato farmers agreed to participate in the on-farm varietal trials, enabling researchers and breeders to incorporate gender into their work.

Some challenges curtailed the incorporation of gender into the breeding program. In the beginning, most stakeholders, especially the breeders, were reluctant to embrace gender. However, over time they came to appreciate this aspect, because research allowed them to identify specific consumer preferences of men and women. Previously, there had been low adoption of some sweetpotato varieties, like the orange-fleshed ones, and the promoters and researchers hoped that gender research would help to promote OFSP at the community level.

Although the multifunctional team was expected to have sufficient information on gendered trait preferences; unfortunately, there was insufficient data on traits like aroma and vigorous vine establishment to conduct a meaningful assessment. Another issue was how to handle the several traits that were identified during gender analysis, given that only a few could be included in the product profile. Finally, some of the proposed traits (fibrousness and sweetpotato smell) need further assessment. Therefore, either genomic tools or high-throughput phenotyping protocols will have to be developed to support development of varieties.

## 4 Assessment of approaches and outcomes of gender integration in sweetpotato breeding

### 4.1 Pros and cons of gender integration approaches

There was a progressive evolution of gender integration in mainstream breeding activities (Table 1). According to the gender mainstreaming continuum (IDRC, 2017), the earliest activities such as PVS and PPB (1992 to 2016) were gender sensitive while more recent activities like the Citizen science (Tricot) and gender food mapping study with RTBfoods (2017 to 2021) had more elements of gender transformative research, given its attempt to rectify past exclusion from breeding by focusing more on women’s needs and priorities. For instance, the tricot approach improved inclusivity by allowing more participants to evaluate clones from the breeding program. Participants who were previously hard-to-reach due to mobility and technology access constraints were given an opportunity grow and evaluate new clones and give feedback.

**Table 1:** Evolution of gender integration in breeding activities.

Activity	How gender was incorporated	Advantages	Shortcomings	Adjustments
PVS (farmers selecting segregating materials)	Before, farmers were considered a homogenous group Later sex disaggregation was included in social analysis Farmer-farmer visits and vine exchanges	Contributed to considering end-users’ preferences and provided a foundation for further social inclusion	Not gender sensitive Analysis and feedback were not sex-disaggregated Only target farmers	Sex -disaggregated farmer selection Sex and age considered at data analysis
PPB (farmers evaluating segregating material)	Before, farmers were considered a homogenous group Later, gender was included in social integration. Farmer-farmer visits and vine exchanges	Contributed to considering end-users’ preferences and was a foundation for further social inclusion and Gender-aware	Not gender-sensitive Analysis and feedback not sex-disaggregated Only target farmers	Sex-disaggregated farmer selection Sex and age considered at data analysis
Choice experiments (AbacusBIO)	Extensive consultation with gender specialists in design Socially-inclusive, diverse respondents – intersectional identities Collected data on gender trait preferences across value chain	Value chain focus Economic selection index for advancing genotypes across the value chain Gender-sensitive	Untargeted questionnaire Reporting had limited gender analysis.	Adopted the use of economic selection Indices
Citizen science involving mass volunteer participation in research	Vines were delivered to households to reduce mobility and access to technology constraints Farmers allowed to use their own farming and cooking practices Local languages are used to communicate Participants received feedback on results from study	Targets participants with intersecting identities Enabled participation of previously hard to reach categories Cost effective More customized priority setting Lighter response burdens for participants Gender responsive	High initial cost and technical requirements	Started using specialized data tools
Social survey research/value chain analysis/gendered food mapping	An adapted gender dimensions framework used for the research tool development (Rubin, 2011) Roots were delivered to local communities Respondents used their own cooking practices Interpretation to local language provided	Some intersectional identities considered Light response burden Consumer-targeted priority setting Complementarity among activities Promoted inter-disciplinary approach Elements of gender-transformative research	Took time to build rapport and cohesion among multiple disciplines	More intersectional identities considered* Deeper gendered analysis on preferred traits* Individual expression facilitated through voting*
Use of G+ Tools for consumer or product profile assessments	Added an extra layer of gender analysis on PP G+ PP tool applied to gendered PP to further assess gender responsiveness of identified traits	Exante assessment of potential gender harm or benefit of a trait Identification of strategies to accompany traits likely to cause gender disparity Allows for multi-disciplinary discussions Allows for shared experience among stakeholders Final consensual scores after discussion are more rational and informed	Initially tool difficult and time-consuming to use Evidence to complete the tool may not exist or may be limited	Used by product design team Gender impact scale still needs to be improved Data analysis at collating results requires improvement
Farmer-managed or small-scale, artisanal seed production (CSPs)	Women represented in leadership roles Both husband and wife are included as beneficiaries Farmer-farmer visits and vine exchanges Formal registration of commercial seed producers into co-operatives or associations	Gender-aware Informs breeders of seed traits relevant to seed producers Inter-disciplinary activity Builds business mindset and visibility Identified market-preferred varieties Facilitates channels for cleaning materials Identification of best fit varieties	High investment costs like irrigation, mini-screenhouses, access to water source excludes women Registration requirements exclude some social groups	CSPs were empowered when registered as cooperatives

*These adjustments executed for potato and sweetpotato in Mozambique.

Although the gender food mapping study was gender transformative, the approach required time to build rapport and cohesion among multiple disciplines. All traits prioritized from the findings from the various approaches can be assessed for gender and livelihood assessment using the G+ Product Profile Query tool, enabling robust gender analysis. However, this tool requires evidence to complete which sometimes may be a draw back.

### 4.2 Changes in breeding process and practice after gender learning

In the multidisciplinary, gendered food mapping study, trade-offs between the disciplines led to omission of some important gender issues at the data collection analysis and inference stages. The study generated a lot of data which needed to be interpreted in a useful way to the breeder. By default, the breeder was designated as the product champion, who was briefed of the findings from various members of the interdisciplinary team. After several iterations, the findings were consolidated for joint conclusions and implications for research and future perspectives.

Gender integration got a breakthrough when the breeders realized how much women affect the choice of sweetpotato varieties grown and how much some sweetpotato traits can make a difference to women’s well-being. This is summarized by the CIP sweetpotato breeder in Uganda:

*“When I got to see how women and sweetpotato were interacting …. I knew that there was no way we were going to succeed without considering traits that women found dear”* Dr. Reuben Ssali, sweetpotato breeder, CIP.

This perspective led to the re-engineering of the PQTs in the breeding pipelines. The larger plot sizes further enhanced this change. The versatility of the crop also was a contributor as explained below:

*“Sweetpotato is the most versatile RTB crop in terms of utilization, because it can be used in a wide range of end-user products. From food on the table, it can also be processed and used as feed… We need to take care of the needs of end-users including women, men and children …. This is when I felt that this would help get products that can be adapted and adopted and utilized.”*- Dr. Bernard Yada, sweetpotato breeder, NACRRI.

In a product advancement meeting led by an economist, breeders equipped participants with basic breeding knowledge. This enabled the interdisciplinary team to integrate social and gender aspects, among other considerations, in the process of selecting varieties to advance for release, as guided by a gender-intentional product profile (Table 2). This interdisciplinary approach allowed for better integration of end-user preferred traits in breeding.

**Table 2:** A gender-intentional target product profile for OFSP in East Africa.

Trait type	Trait	Scale	Desirable score	Trait requirement	Improve trait	Thresh-old trait	*Gender score (0–2)
Color	Skin color	1 to 9	2 & 7	Nice to have			1
Flesh type	Flesh color	1 to 9	6, 7 & 8	Essential	Y		1
Processing traits	Optimal cooking time (250 g)	Minutes	20	Essential	y		2
	Boiled sweetpotato uniformity of cooking	0 to 10	10	Nice to have			2
	Raw sweetpotato ease to peel	0 to 10	2	Nice to have			2
Consumption traits	Dry matter content	%	32	Essential		Y	2
	Boiled sweetpotato mealiness	0 to 10	7	Essential	y		2
	Boiled sweetpotato hardness	0 to 10	6	Essential	Y		2
	Boiled sweetpotato sweet	0 to 10	7	Essential	Y		2
Nutritional traits	Beta carotene content	mg/100, DW	20	Nice to have			2
	Iron content	mg/100, DW	3	Nice to have			2
	Zinc content	mg/100, DW	2	Nice to have			1
Yield traits	Storage root yield (rainfed)	t/ha	15	Essential	Y		1
	Roots per plant	Number	3	Nice to have			
	Root size	1 to 9	3	Nice to have			1
	Root shape	1 to 9	2 & 6	Essential	Y		2
	Harvest index	%	40	Essential	Y		1
Agronomic traits	Vine vigor	1 to 9	6		Y		1
	Plant growth habit	1 to 9	>5				0
	Vine yield	t/ha	20	Essential	Y		1
Disease traits	Alternaria resistance	1 to 9	3	Essential	Y		1
	SPVD resistance	1 to 9	3	Essential	Y		1
Insect traits	Sweetpotato weevil damage	1 to 9	3	Essential		Y	1
	Caterpillar resistance	1 to 9	3	Essential		Y	1
Maturity	Early to intermediate (100–130 days)						
Production/multi-plication traits	Vine survival	%	70	Essential		Y	2
Key competitive products	Kabode, Alamura, Terimbere, KENSPOT 4 and NASPOT 8						

*Gender score:

0 = not targeted.

1 = significant (gender-aware or gender-responsive).

2 = principle (gender equality is the main objective of this trait).

### 4.3 Breeding outcomes and impacts related to gender equity

The gender-responsive breeding created three varieties: NASPOT 11 which was released in 2010, NASPOT 12 O and NASPOT 13 O released in 2013. These outcomes are mostly attributed to PPB and PVS combining the strengths of farmers and researchers (Gibson et al., 2008; Mwanga et al., 2016). The PPB started in 2003 and three mixed-sex farmer groups in the districts of Luwero, Kiboga and Mpigi participated right from the early stages of breeding. By the third year the participating farmers were already eating the roots and selling them in the fourth year. A comparison between NASPOT 1 (a released variety), NASPOT 11 and Dimbuka (a landrace) by 44 farmers (31 women, 11 men) revealed that NASPOT 11 outperformed the other two on the key agronomic attributes. This accelerated both the breeding process and varietal adoption.

CIP has developed a manual to guide evaluation of sweetpotato trials during PVS. Assessment is sex-disaggregated, where men and women grade traits using color cards for “not acceptable,” “more or less acceptable,” “clearly acceptable” (Gruneberg et al., 2019). The breeding program worked with 100 farm households for two seasons in five districts (Isingiro, Buyende, Rakai, Oyam and Kabale) for the PVS trials (Mwanga et al., 2016). The breeders intended to recruit equal numbers of males and females, but women were more willing to participate, and they outnumbered men on both the on-farm trials and the palatability tests. NASPOT 12 O and NASPOT 13 O were outcomes of PVS. The two varieties were reported to have higher storage root and biomass yield, harvest index, and sweetpotato virus disease (SPVD) resistance compared to Dimbuka-Bukulula. However, at first entry into the market, the adoption was pushed by high demand for vines and at this stage men displaced women as the main beneficiaries, as men were attracted by the business opportunity of selling vines. This could have been mitigated by having a defined gender strategy to include men from planning to marketing and avoid displacing women as evidenced elsewhere. In Mozambique, an initiative to commercialize sweetpotato resulted in women retaining dominancy in the roots chain due to inclusive strategies. For example through training and advocacy, men were encouraged to allow their spouses to engage in commercial activities (Mayanja et al., 2022).

## 5 Discussion

### 5.1 Good practices

Good practices contributed to the evolution of gender-responsive breeding, notably the progressive change from women vs. men to comparisons between social groups and intersecting identities among value chain actors. This led to a more nuanced approach to discern the different preferences of male and female actors along the value chain, thus widening the scope of inclusivity.

Transition from single to multidisciplinary approach, and later to an interdisciplinary one, led to more integration (Troullaki et al., 2021). This enhanced learning among the various disciplines for a common good. For example, gender specialists obtained a better understanding of biological and food sciences related to breeding from other team members. Capacity development and hands-on support in using new methods and tools allowed the social scientists to improve their understanding of food science and breeding. There was constant hands-on learning by all team members, especially on the cross-functional teams where not only academic disciplines, but other actors in the value chain were included. Interdisciplinary and hierarchical differences were reduced which allowed for mutual respect among disciplines and enabled equal participation in activities. This improved the social relations among team members.

Breeders were directly involved in the tasks of the different disciplines, from study design all the way through to data analysis, which enabled them to appreciate the results of research led by different disciplines. Breeders are now championing the value of working with multidisciplinary teams and integrating gender in routine decision making for the product development pipelines.

As a result, there is more value placed on the data from the PQTs (lab values) and breeders wait for the data even though it takes more time for the output to be delivered. This shows an institutional change resulting in breeders designing projects differently. Teams must be composed differently, and time allocated differently, to allow for more robust product profiling where significant gender-transformative changes are expected to occur.

The RTBfoods project continuously added to the set of existing phenotyping tools (especially for biochemistry and biophysical sciences). The new tools enabled us to quantitatively measure the PQTs, which enabled breeders to include them in the product profile.

### 5.2 Lessons

Dissection of the traits identified during gender food mapping helped to reveal embedded attributes within what was previously considered as ‘dry matter’. As a result, we now assess and measure four traits instead of just dry matter alone (mealiness, firmness, water absorption and optimal cooking time). A combination of methods clearly revealed the important traits, which were identified right from the SoK through to consumer diagnosis. The important traits were then included in the target product profiles.

Applying the G+ Product Profile Query tool (Ashby and Polar, 2021; CGIAR, 2021) as a first step to test the gender responsiveness of traits led to a deeper understanding of the traits. For example, breeders realized that traits like yield and smooth skin could potentially cause gender disparities and displace women in commercial nodes of the value chain. Consequently, mitigation strategies were designed to address these issues at varietal dissemination. For instance, before releasing a new variety we plan to conduct demand-creation trials and prepare information packages targeting female value chain actors to guide marketing, good agronomic and post-harvest handling practices for the new variety.

Among the major challenges faced were the gender data collection gaps. As a result, our first publication (Mwanga et al., 2021) focused mostly on food science with limited depth in gender enquiry. Another obstacle to engendering the sweetpotato product profile is that while sweetpotato flavor is considered highly desirable by women, it is chemically complex and expensive to measure.

### 5.3 Recommendation

In retrospect, we found that creating a buy-in for all the multidisciplinary team members should have been one of the first steps taken in this investigation. Giving all team members a shared vision would have greatly eased the research. Our future goal is to scale gender integration into other national breeding programs and to extend this process to other areas of the breeding pipelines such as marketing and seed systems.

We recommend that breeding teams elsewhere establish multi-functional teams. An inclusive vison of gender would capture the needs of men and women all along the sweetpotato value chain. This requires understanding how social identities interact to exclude people from certain activities because of their gender. Gender research is a rigorous undertaking that requires expertise, time, money, and adequate preparation. This requires establishing interdisciplinary research teams which are fully engaged for joint decision making throughout the entire product advancement process.

## Data availability statement

The original contributions presented in the study are included in the article/Supplementary material, further inquiries can be directed to the corresponding author.

## Author contributions

RS, SM, MN, BY, and VP contributed to conception and design of the study. JM and MN organized the schematics and figures. RS, ST, DM, JM, and SM wrote the first draft of the manuscript. IB, DM, BY, JO, RM, and LF wrote sections of the manuscript. All authors contributed to manuscript revision, read, and approved the submitted version.
