# Supplementary material for: Gender mainstreaming in sweetpotato breeding in Uganda: a case study
Source: Front Sociol. 2023 Dec 15;8:1233102. doi: 10.3389/fsoc.2023.1233102 (PMC10757364; doi:10.3389/fsoc.2023.1233102)
Supplement: Supplementary file 1 [file Data_Sheet_1.docx]

**SCOPE OF ACTIVITIES, REPORTS AND DELIVERABLES**

**Project Title:** **“*Elaboration of case study integrating gender into breeding objectives and decision”***

1. ***Background:***

The CGIAR GENDER Impact Platform seeks to share knowledge widely about common elements and lessons learned in the experiences integrating considerations of gender differences into plant breeding and thereby improving gender integration in breeding programs, with the ultimate goal of advancing gender equality. In August 2022, CIP in close collaboration with Gender Responsive Researchers Equipped for Agricultural Transformation (GREAT) released a call to gather - from cases and experiences submitted by different organizations - an overview of the state of the art of gender-responsive breeding and to promote knowledge-sharing among breeding programs.

Integrating social welfare objectives in plant breeding is a rapidly growing approach on a global scale and particularly in research for development, re-shaping the technical options, goals and intended impact of breeding programs. Improving the benefits delivered to rural women by implementing gender-responsive breeding is one facet of this change, generating new methods and learning. Gender-responsive breeding presents a unique opportunity to develop targeted interventions to improve outcomes for different stakeholders based on their needs and interests. This targeted approach to breeding can, in particular, better address the goals, needs and concerns of women.

Learning distilled from the identified cases will be applied by the CGIAR GENDER Impact Platform to inform ongoing projects and future integration of gender in breeding.

1. ***Objective of Partnership:***

Through this partnership, CIP formalizes the selection of the Program Participant’s experience by an expert panel to write a short, structured case study.

The case study will follow the guidelines described in Section 4 below. Experiences documented will be published for knowledge sharing, teaching, and self-instruction purposes.

The resulting case study will be a journal-length paper authored by the implementing team. The authors will receive guidance for systematization from CIP and the GREAT team.

1. ***Main Activities and commitments:***

The Program Participant shall implement the activities and furnish all Project milestones and deliverables for each case study separately as set forth here in Annex 4 and Annex 5:

| **Activity** | **Description*** | **Responsible** | **Dates to complete activity** |
| --- | --- | --- | --- |
| Activity 0 | Selection of experience submitted by the Program Participant in response to the call released in August 2022. | CIP and Expert Panel | November 2022 |
| Activity 1 | Individual consultations, elaboration of case study outline and first draft of case study. | Program Participant | 16^th^ December 2022 |
| Activity 2 | Review of first draft case study. | CIP and Expert Panel | 16^th^ – 20^th^ December 2022 |
| Activity 3 | Post-review reflection and incorporation of adjustments in final version. | Program Participant | 4^th^ January 2022 |
| Activity 4 | Second review of case study draft | CIP and Expert Panel | 4 - 11^th^ January 2022 |
| Activity 5 | Elaboration of final version of full-length case study ready for publication and Power Point synopsis of case. | Program Participant | 20^th^ January 2022 |
| Activity 6 | Editorial review and feedback for submission | CIP – communication support | January 30^th^ |
| Activity 7 | Authors webinar presentation of case (presenting PPT Synopses) | Program Participants | TBD |

**Each case study (draft and final version) should be structured in line with the guidelines in Section 4.*

Furthermore, the Program Participant will provide CIP with such information as is necessary to allow CIP to comply with its reporting obligations with the CGIAR (Sponsor) under the Prime Award.

1. **Guidelines for the elaboration of the case study:**

# Guidelines for Case Studies of Gender-Responsive Plant Breeding

## **Background**

The CGIAR Gender research team, led by the International Potato Centre (CIP), and the Great program, led by Cornell University, have conducted an Open Call inviting submissions of brief summaries of experience with gender-responsive breeding. Selected responses to the Call are eligible for a grant for writing a case study, each in the format of a journal article. Case studies will be published as a journal special issue of a book.

## **Purpose of the Guidelines**

There are two parts to these Guidelines. The first part describes components of the plan of work and schedule that authors (grant recipient or Program Participant) will undertake once a grant is accepted. The second part of the Guidelines covers the structure to be used by authors when writing a case study.

# Plan of work and schedule

Grant recipients undertake the following commitments:

1. Write a completed Case Study of gender-responsive breeding following the structure and format for writing a Case Study supplied
2. Contribute to a 60-minute, virtual inception meeting with fellow grantees/ authors and one or more Gender Team members to discuss the objectives of the case studies; what is expected of authors; what is understood as gender-responsive breeding, and what kinds of lessons cases might portray. This group meeting will also cover any logistic questions.
3. Participate in a virtual consultation to discuss their outline and the evidence they plan to use in the paper.
4. Participate in at least one individual consultation, submit case study outline and the first draft of the full-length Case Study as per the schedule for review and feedback
5. Incorporate feedback and submit a second draft of the Case Study for editing as per the schedule
6. Review editing and submit final paper along with a Power Point Synopsis of case
7. Present a PowerPoint synopsis of the Case in a webinar and contribute to the discussion of lessons drawn from the Cases presented. Date to be determined.

# II. Guide for writing a Case Study of Gender-responsive plant breeding

The objective of the Case Studies is to analyze the experience of implementing gender-responsive breeding to answer the question: What can we learn from effective or successful experiences as well as ones that have not worked as well as expected? To become more gender-responsive, a breeding program needs to answer two questions:

- Are there differences among our end-users associated with gender, that might affect our breeding objectives or impact?
- If the answer is “Yes” what can we do about it?

A program that has addressed these questions scientifically using evidence and finds there is no feasible action available, is still considered gender-responsive. In other words, breeding programs do not have to develop “varieties for women” to become gender-responsive, but they do need to address these two questions responsibly.

A Case study tells the story of what happened when these questions were addressed, analyzes the obstacles encountered and how they were tackled, identifies opportunities to improve gender responsiveness, and draws lessons from the experience.

The completed Case study should be approximately 5,000 words including References, Figures, and Tables, and formatted as a journal article. Please include references throughout where appropriate.

All second drafts will receive professional editing. Each author should submit their final Case Study to the Selected Journal. For specific formatting guidance, see Journal specifications.  **Each Case Study should make use of the content included in their Response to the Open Call.**

## Structure of the Case Study

1. **Title, authors, institutional affiliates, acknowledgments, abstract**

**INTRODUCTION**

1. **Why did the breeding initiative pay attention to gender?**

Explain the problem or the need that attracted attention to gender issues and why this motivated the breeding to become more gender-responsive.

- **Gender relations in the crop**: provide a context for the problem by describing relevant aspects of gender relations and any other social, intersectional aspects of how the crop(s) is grown and used. For example, describe how gender and social differences affect yields and productivity, labor use or drudgery, land ownership, access to purchased inputs, information, transportation, markets and technology, access to new varieties and seed, marketing arrangements, processing, how the crop -- or income from it, is distributed in the household or community.

1. **Context**

Describe the types of organizations and actors involved; the geographical scope of the plant breeding program (country or countries or regions); the crop or crops; size and composition of the breeding team; chief characteristics of target beneficiaries (producers and others in the value chain)

**ANALYSIS**

1. **What research or other sources of information on gender were generated?**

Diagram a timeline of gender research activities

Explain who did what in gender research and when with reference to the timeline

Explain what data were collected, when, and why? How were data used?

1. **How did attention to gender in**fl**uence the breeding initiative? Tell the story of what happened**

**Note:** This is an important section of the case study. Aim for a detailed narrative of who did what, when, where, and why. How did breeding objectives or practice change with the aim of becoming more gender-responsive? What opportunities arose that made it feasible to integrate gender issues into breeding? Or, if your Case Study shows that gender issues were raised but did not result in changes to breeding objectives or practice, explain what led to this result. What obstacles made it difficult to get attention for gender analysis? Were there constraints that affected breeders’ receptivity to findings from gender analysis? What difficulties were overcome, and how? Were there insurmountable obstacles and why?

- Diagram a timeline of key events and especially, decisions in the breeding process.
- On the timeline, highlight key points in time when gender research engaged with decisions made by breeders or others involved in developing and disseminating varieties and seed.
- Your story should tell who did what (gender researchers, breeders, agronomists, community leaders, politicians, farmers, other institutions, gender researchers, etc.) with reference to your timeline.
- Focus on human interactions in telling your story without being personal. Who and what kinds of people made the process easy or difficult? Who provided leadership? What motivated the different people involved to consider being more gender-responsive? Were some people resistant? Why? How did this change, if it did? How and why did some people disagree? Did they change their minds? Was there a process of consensus -building and how did it evolve?
- Consider the changes listed in Question 13 in your Response to the Open Call or any other changes you identify for your Case, and make sure your narrative tells the story of how any of these changes came about

1. **Methods and approaches: advantages and shortcomings**

Refer to Question 19 “Did the Case involve any of these activities?” of your Response to the Open Call.

- Make a Table listing each of the activities involved in Question 19. Add to the Table any other activities you think should be included in your Case
- Note in the Table whether an activity involved specific attention to gender.
- Write a short description of what was done in your Case for each activity, method, or approach in your Table, and where relevant, explain how attention to gender was incorporated. Did some approaches have a special advantage? Did any approach make incorporating gender easier or more difficult? What adjustments or adaptations were made in your Case to bring gender analysis into play?

1. **What in the breeding process and practice has changed as a result of learning about gender?**

For topics, refer to Question 13 in your Response to the Open Call. This section should pull together an overview of the obstacles, opportunities and changes realized that you’ve already recounted in your narrative for Section V above. This section should highlight any bottlenecks that held up progress or elements that made the process go smoothly.

1. **Breeding outcomes and impacts, especially those related to the impact on gender equity**

Please make sure that if you show new varieties were released as a result of the activities you’ve narrated earlier, or if there was a follow-up on the adoption of varieties that you clarify when these took place in reference to the timeline

**DISCUSSION**

1. **Good practices**

Refer to Question 18 “How well do these statements describe practice…?” in your Response to the Open call. Use each item 1-8 relevant to your Case. Add other good practices from your case to the list. Use each “good practice” as a subheading and write a short paragraph about each one, focusing on who did what and why the practice is desirable.

1. **Lessons**

What went well? What worked?

What went wrong? What mistakes were made?

What obstacles or bottlenecks need to be resolved?

With hindsight, what do you think could be done differently and why?

What opportunities exist for further work and how would you approach them?

What would you recommend to others starting out who might encounter similar opportunities or face similar difficulties to the ones in your Case?

1. **References**
2. **Figures and Tables**

Please include the following. Add others relevant to your case.

**Figure 1 Timeline of Gender Research in the Case of …..**

**Figure 2 Timeline of Key Events and Breeding Decisions in the Case of….**

**Table 1 Methods and Approaches in the Case of….**
